# Supplementary figures and images for: Fasciclin 2 engages EGFR in an auto-stimulatory loop to promote imaginal disc cell proliferation in Drosophila
Source: PLoS Genet. 2022 Jun 6;18(6):e1010224. doi: 10.1371/journal.pgen.1010224 (PMC9203005; doi:10.1371/journal.pgen.1010224)

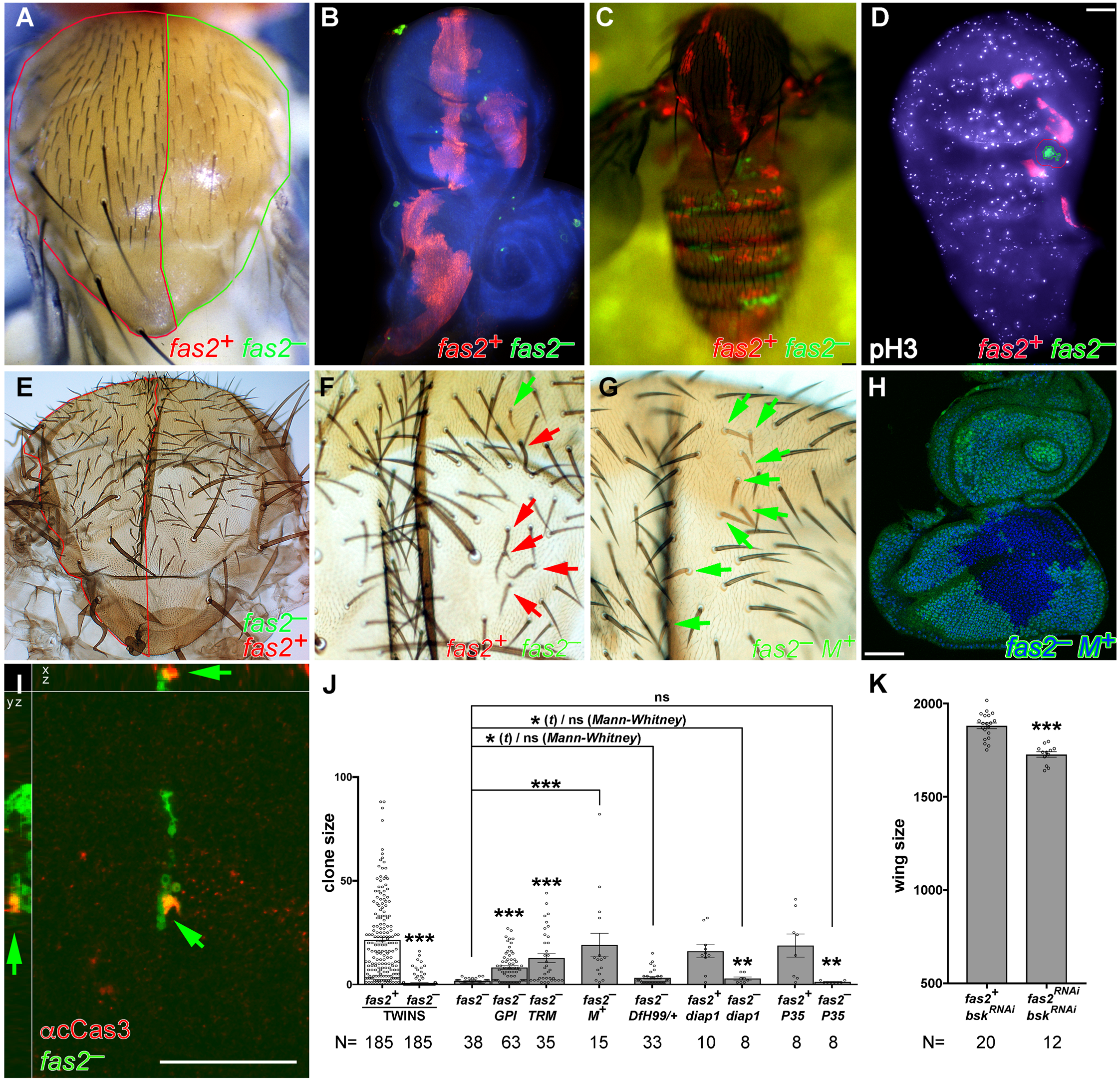

Supplement: S1 Fig — (A) Gynandromorph (y fas2eB112/R(1)2) displaying a Fas2-deficient right side (y fas2eB112, labeled with the yellow marker, green outline). (B) Coupled-MARCM Fas2-deficient clones (fas2eB112, fas2– labeled with GFP) and their control Fas2-normal twins (fas2+ labeled with mtdTomato) in a wing imaginal disc at puparium formation. The Fas2-deficient clones are missing or consist of single cells, but their fas2+ twins can be larger than average WT clones, reminiscent of the Minute effect. (C) fas2– coupled-MARCM clones (labeled with GFP, green) show a grow deficit in imaginal disc derivatives compared to their control twins (mtdTomato, red) but do much better in abdominal histoblasts. Compare the number and size of fas2– clones (GFP) with that of their twin fas2+ controls (mtdTomato) in notum vs. abdomen. The picture corresponds to an adult just after eclosion. (D) Coupled-MARCM fas2eB112 clones (GFP) and twin control clones (mtdTomato) in a wing disc stained with anti-pH3 antibody to reveal mitosis. The red outline marks the rim of 2–3 cells around the Fas2-deficient clone (used to quantify the mitotic index). (E) Notum of an adult individual with the whole left heminotum covered by control clone territory (sn, fas2+ outlined in red), but without any detectable Fas2-deficient twin (which would have been labeled with y f36a, fas2—). (F) At least some Fas2-deficient cell clones survived in the adult. The picture shows a y fas2eB112 f36a clone (green arrow) and its sn3 control twin (red arrows point to sn3 bristles). The clone was induced in 2nd instar larva. (G) y fas2eB112 f36a M+ clones in a Minute heterozygous background are able to grow normally and differentiate epidermis in the adult (green arrows point to y fas2eB112 f36a chaetes). (H) Eye clone deficient for Fas2 induced in a Minute genetic background. fas2eB112 M+ null clones (without GFP) could grow more normally in the M–/M+ heterozygous background (labeled with Ubi-GFP). Genotype: y fas2eB112 FRT18A/Ubi-GFP M( [file pgen.1010224.s001.tif]

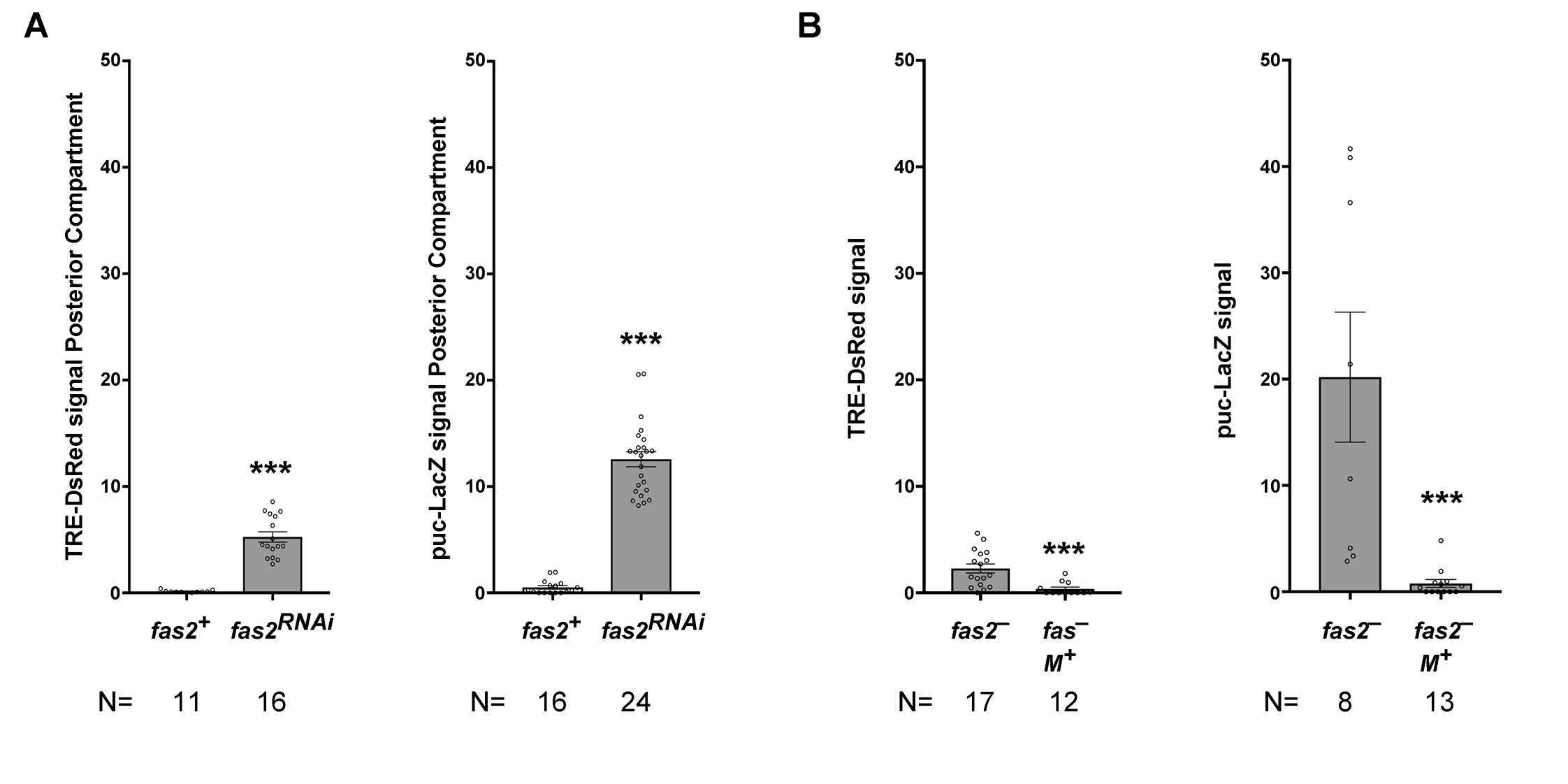

Supplement: S2 Fig — (A) Left, quantification of TRE-DsRed signal in en-GAL4 UAS-GFP/TRE-DsRed (fas2+) and en-GAL4 UAS-GFP/TRE-DsRed; UAS-fas2RNAi#34084/+ (fas2RNAi) wing imaginal discs. The TRE-DsRed signal was amplified using an anti-RFP antibody, and the signal in the anterior compartment was subtracted to the signal in the Posterior compartment in each disc to normalize for differences in staining. Right, quantification of puc-LacZ signal in en-GAL4 UAS-GFP/+; puc-LacZ/+ (fas2+) and en-GAL4 UAS-GFP/+; UAS-fas2RNAi#34084/puc-LacZ (fas2RNAi) wing imaginal discs. The signal in the anterior compartment was subtracted to the signal in the Posterior compartment in each disc to normalize for differences in staining. N is number of wing imaginal discs. (B) Left, quantification of TRE-DsRed signal in fas2eB112FRT19A; TRE-DsRed/; Tub-GAL4 UAS-GFP/+ MARCM null cell clones (fas2–) and fas2eB112 FRT18A; TRE-DsRed/+ Minute+ null cell clones (fas2– M+) induced in fas2eB112 FRT18A/Ubi-GFP M(1)Osp FRT18A; TRE-DsRed/+ wing imaginal discs. The signal in the background was subtracted to the signal in the clone in each disc. Right, quantification of puc-LacZ signal in fas2eB112FRT19A; Tub-GAL4 UAS-GFP/puc-LacZ MARCM null cell clones (fas2–) and fas2eB112 FRT18A; puc-LacZ/hs-FLP Minute+ null cell clones (fas2– M+) induced in fas2eB112 FRT18A/Ubi-GFP M(1)Osp FRT18A; puc-LacZ/hs-FLP wing imaginal discs. The signal in the background was subtracted to the signal in the clone in each disc. N is number of clones. (TIF) [file pgen.1010224.s002.tif]

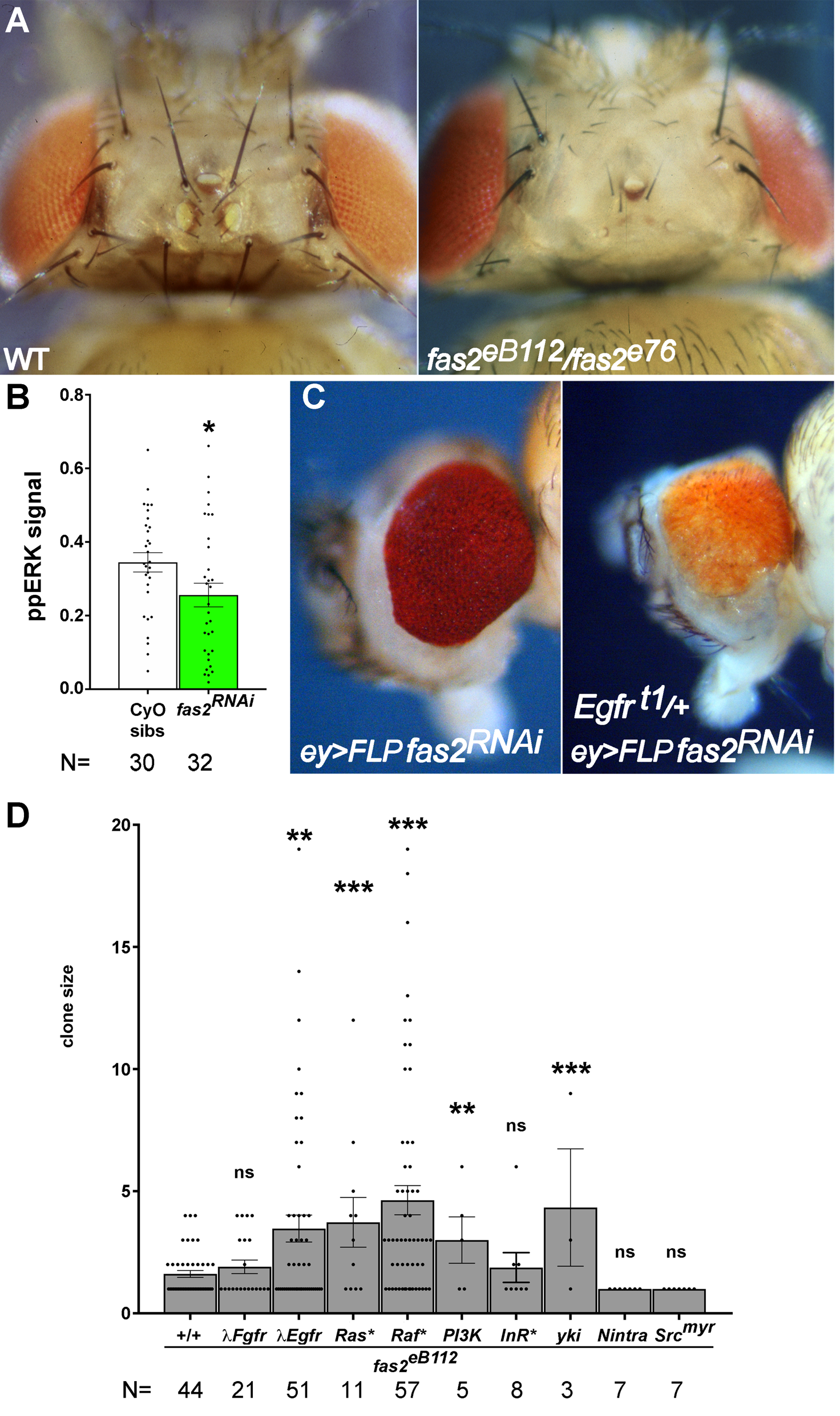

Supplement: S3 Fig — (A) Left, WT adult head. Right, the hypomorphic fas2eB112/fas2e76 combination displayed the absence or size reduction of ocelli, as well as loss of bristles in the dorsal head, an alteration reminiscent of Egfr torpedo alleles (Egfrtop). (B) Quantification of fluorescent ppERK antibody signal (ratio of pixels with a signal level higher than 100, out of 255 levels) in the eye disc of ey-driven fas2RNAi (#34084) FLP-OUTs (green bar) and their control CyO siblings (white bar). N is number of eye imaginal discs. (C) Left, ey-driven fas2RNAi (#34084) FLP-OUT. Right, ey-driven fas2RNAi (#34084) FLP-OUT in an Egfrt1/+ heterozygous background. (D) Suppressors of the fas2-null MARCM-clone phenotype in the adult. MARCM fas2eB112 clones labeled with yellow and forked were induced in combinations expressing UAS-insertions for components of different growth signaling pathways. Expression of activated components of the EGFR signaling pathway (RasV12, RafGOF and PI3K - Dp110-) and over-expression of Yki produced a significant correction in the size of fas2—clones in the adult notum. In contrast, expression of activated-FGFR (λHtl), which shares most downstream effectors with EGFR, activated-InR (InRR418P), activated-Notch (NINTRA) and myristoylated-Src did not cause a significant suppression. Clone size is number of marked microchaetes. N is number of clones. (TIF) [file pgen.1010224.s003.tif]

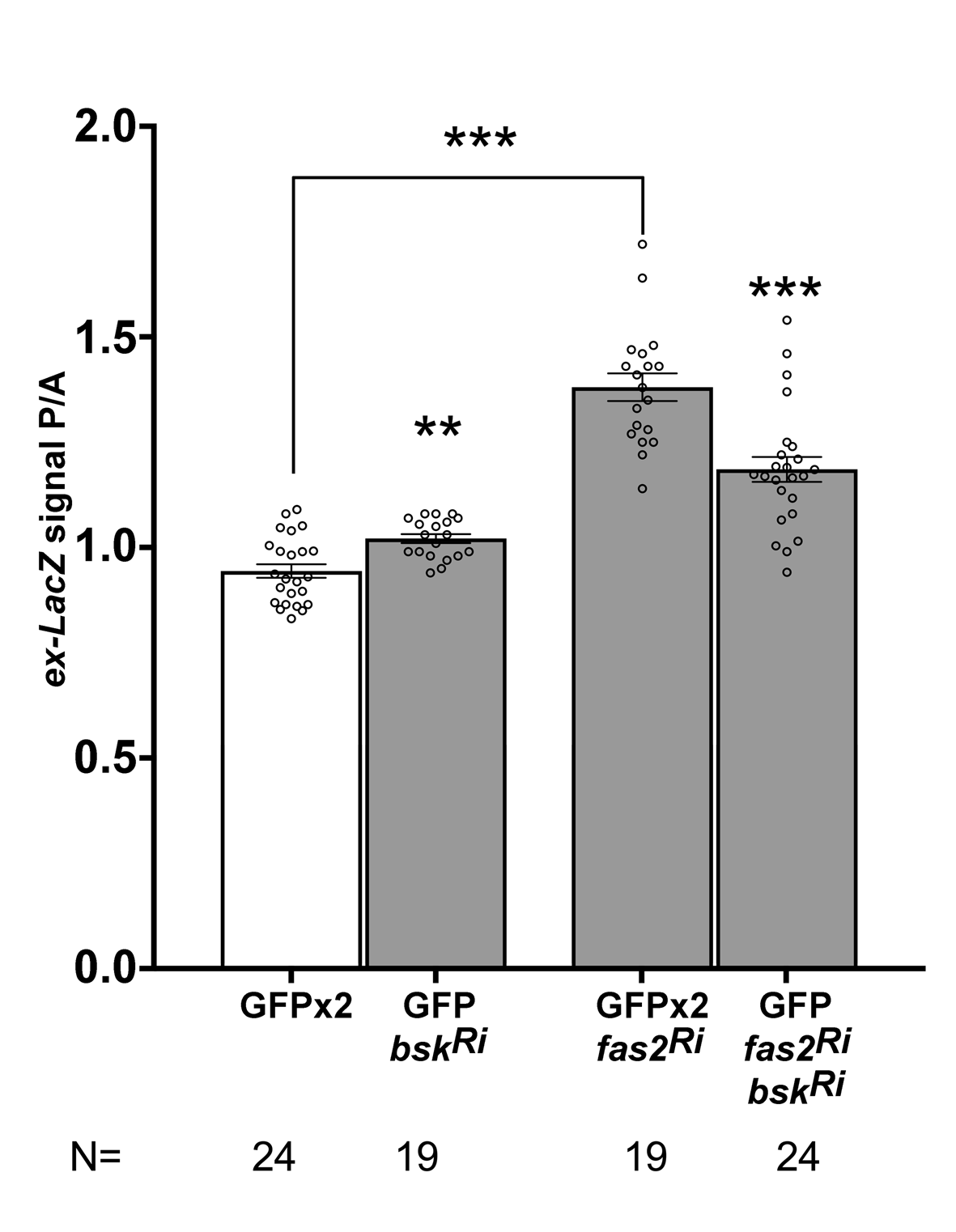

Supplement: S4 Fig — Quantification of ex-LacZ reporter expression in en-GAL4/+; fas2RNAi#34084 wing imaginal discs. The intensity of expression of the ex-LacZ reporter (measured as grey average in the red channel) is similar in the anterior and posterior compartments of each control wing imaginal disc (giving a posterior/anterior signal ratio close to 1.0). Expression of UAS-fas2RNAi (#34084) in the posterior compartment causes a strong increase of ex-LacZ expression compared with the anterior compartment in the same disc (ex-LacZ P/A signal ratio). While inhibition of the JNK pathway (UAS-bskRNAi, #32977) in the posterior compartment slightly increases ex-LacZ P/A signal ratio, it strongly suppresses the increase caused by the inhibition of Fas2 expression. N is number of wing imaginal discs. (TIF) [file pgen.1010224.s004.tif]

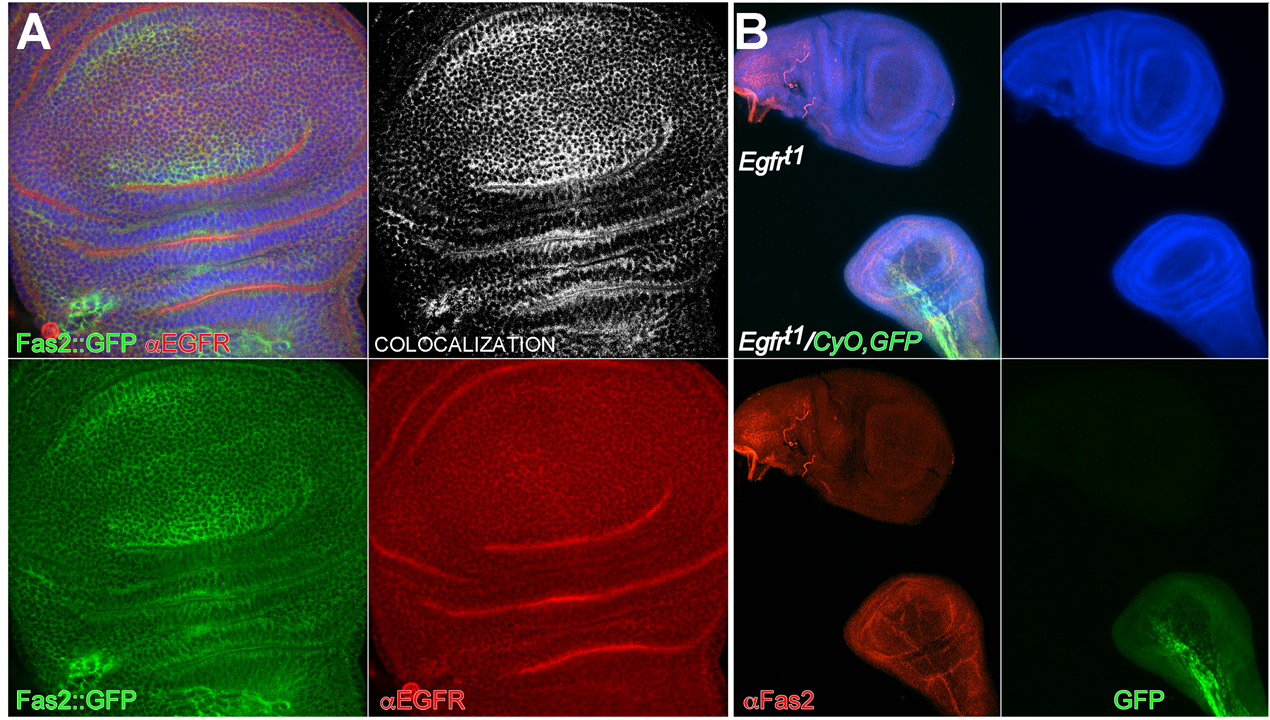

Supplement: S5 Fig — (A) Fas2 and EGFR are expressed by all cells in imaginal discs. A Fas2::GFP protein trap [13] shows colocalization with EGFR (ImageJ Colocalization plug-in, Pearson´s correlation: 0,3146). (B) Homozygous Egfrtop1 imaginal discs showed a lower expression of Fas2 (red channel, labeled with the anti-Fas2 1D4 antibody) than the imaginal discs from their heterozygous siblings (Egfrtop1/CyO, GFP). (TIF) [file pgen.1010224.s005.tif]

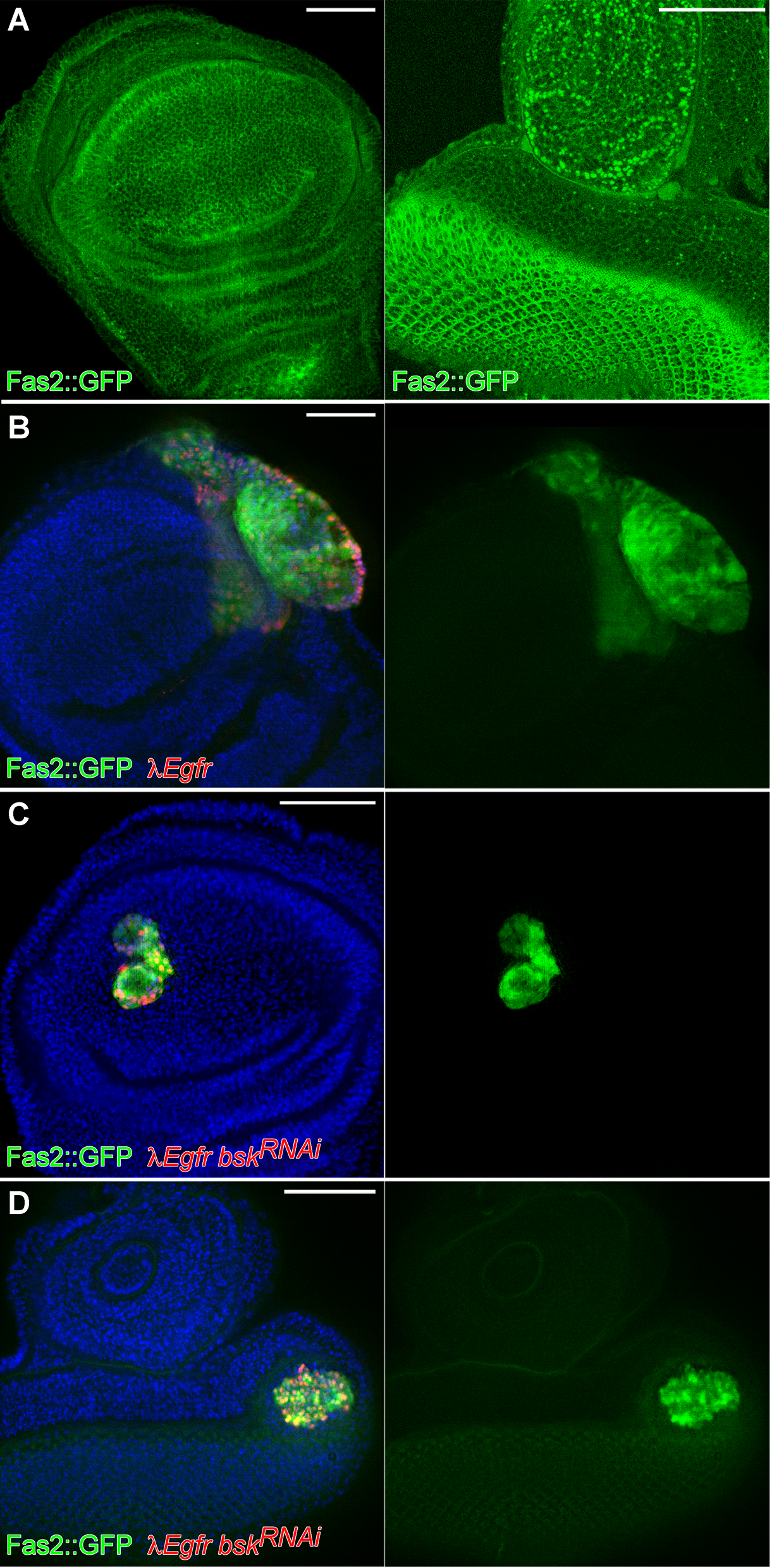

Supplement: S6 Fig — (A) A Fas2::GFP protein trap line shows expression of Fas2 in all cells of imaginal discs, with a maximum in differentiating retinal cells. (B) FLPOUT UAS-LacZ clones (red signal) expressing activated-EGFR (λEgfr) display a dramatic increase in Fas2::GFP expression. Note that the saturation of the Fas2::GFP expression prevents the visualization of the normal Fas2::GFP expression in the other cells of the wing disc (compare to Fig 7A which is stained with the 1D4 antibody that only recognizes the TRM isoforms of Fas2). (C) Wing imaginal disc FLPOUT UAS-LacZ clones (red signal) expressing activated-EGFR (λEgfr) plus bskRNAi314767 display a Fas2::GFP signal similar to activated-EGFR clones. (D) Eye imaginal disc FLPOUT UAS-LacZ clones (red signal) expressing activated-EGFR (λEgfr) plus bskRNAi#31476 display a similarly strong Fas2::GFP signal. Note that the saturation of the Fas2::GFP signal only permits a very faint visualization of the normal Fas2 peak of expression in the differentiating retina. (TIF) [file pgen.1010224.s006.tif]
