# Supplementary material for: Fasciclin 2 engages EGFR in an auto-stimulatory loop to promote imaginal disc cell proliferation in Drosophila
Source: PLoS Genet. 2022 Jun 6;18(6):e1010224. doi: 10.1371/journal.pgen.1010224 (PMC9203005; doi:10.1371/journal.pgen.1010224)
Supplement: S1 Text — (DOCX) [file pgen.1010224.s007.docx]

**SUPPLEMENTARY MATERIALS AND METHODS**

**Strains used for coupled-MARCM analysis**

*y QS13F FRT19A/FM7-GFP; QF-ET40 QUAS-mtdTomato/CyO*

*y fas2^eB112^ QS13F FRT19A/FM7-GFP; QF-ET40 QUAS-mtdTomato/CyO*

*y fas2^eB112^ QS13F FRT19A/FM7-GFP; UAS-bsk^RNAi#32977^*

*y fas2^eB112^ QS13F FRT19A/FM7-GFP; UAS-*λ*Egfr/TM6B*

*y fas2^eB112^ QS13F FRT19A/FM7-GFP; UAS-yki/TM6B*

*y fas2^eB112^ QS13F FRT19A/FM7-GFP; UAS-InR^DEL^/TM6B*

*y fas2^eB112^ QS13F FRT19A/FM7-GFP; UAS-InR^418P^/TM6B*

*w hsp-flp Tub-GAL80 FRT19A; Tub-GAL4 UAS-GFP/TM6B*

**Strains for MARCM analysis**

*y fas2^eB112^ sn^3^ FRT19A/FM7-GFP; UAS-GFP; MS1075-GAL4* (used for clones in adults)

*y fas2^eB112^ FRT19A/FM7-GFP; TRE-DsRed*

*w hsp-flp Tub-GAL80 FRT19A; UAS-X* (*UAS-X* represents the different UAS constructs

presented in Results)

*w hsp70-flp Tub-GAL80 FRT19A; puc-LacZ^E69^/TM6B*

*w hsp70-flp Tub-GAL80 FRT18A; TRE-DsRed*

*Strains for FLP-OUT clone analysis*

*y w ey-flp; Act5C-FRTy^+^FRT-GAL4 UAS-GFP/CyO*

*y w hsp70-flp; Act5C-FRTy^+^FRT-GAL4 UAS-GFP/CyO*

*y w hsp70-flp; Act5C-FRTy^+^FRT-GAL4 UAS-GFP/CyO; UAS-fas2^RNAi34084^*

*y w hsp70-flp; Act5C-FRTy^+^FRT-GAL4 UAS-GFP*; *puc-LacZ^E69^/TM3*

*y w hsp70-flp; Act5C-FRTy^+^FRT-GAL4 UAS-LacZ*

*y w hsp70-flp; Act5C-FRTy^+^FRT-GAL4 UAS-LacZ*/SM6a-TM6B/*UAS-bsk^RNAi31476^*

*y w fas2::GFP; UAS-λEGFR*

**Other strains to study fas2– clones in the adult and with the Minute technique**

*y fas2^eB112^ f^36a^ FRT18A/FM7c* (used for clones in adults)

*w sn^3^ FRT18A; hsp-flp* (used for clones in adults)

*w M(1)O^sp^ FRT18A/FM7; hsp70-flp; Dp(1;3)A59*/*TM6B* (used for clones in adults)

*Ubi-GFP M(1)O^sp^ FRT19A/FM7a; hsp70-flp*

*Ubi-GFP M(1)O^sp^ FRT18A/FM7a; TRE-DsRed*

*Ubi-GFP FRT18A/FM7a; TRE-DsRed*

*fas2^eB112^ FRT19A/FM7, GFP; hsp70-fas2^TRM31.2^/TM6B*

*fas2^eB112^ FRT18A/FM7, GFP; hsp70-fas2^TRM31.2^/TM6B*
