# Supplementary material for: Fasciclin 2 engages EGFR in an auto-stimulatory loop to promote imaginal disc cell proliferation in Drosophila
Source: PLoS Genet. 2022 Jun 6;18(6):e1010224. doi: 10.1371/journal.pgen.1010224 (PMC9203005; doi:10.1371/journal.pgen.1010224)
Supplement: Attachment — Submitted filename: Response to reviewers.docx [file pgen.1010224.s008.docx]

**Reviewer #1:** PGENETICS D 21 01398

In this very nice paper, Velasquez et al report a novel function of the Drosophila NCAM homolog Fasciclin2. As NCAM, Drosophila Fasciclin2 is a homophilic cell- cell adhesion protein that as the authors convincingly show is cell autonomously needed to promote cell proliferation in imaginal discs. As validate by a thorough combination of loss and gain of function genetics, and immunohistochemical studies, this proliferative trigger is mediated via the EGF-receptor. Importantly, as the authors demonstrate at the end of their manuscript, the EGF-receptor can physically interact with Fascilin2. Quite interestingly, the authors note that activation of the EGF-receptor leads to an increase in Fasciclin2 expression suggesting the presence of a positive feedback loop sustaining EGF-receptor activation and Fasciclin2 expression.

This paper in particular important as it contrasts previous reports where it was shown that in differentiating Drosophila photoreceptor cells, Fasclin2 and EGF-receptor act in an antagonistic relationship (Mao and Freeman, 2009). As mentioned in the discussion, these two reaction properties may reflect different Fascilin2 concentrations, which will be an interesting question to address in future studies.

The data are documented by beautiful and very clear Figures. Just in Figure5A the authors may consider a revision to show intact, non-tilted brains without any imaginal disc obscuring the imaging.

Very nice and important work showing a new twist in a long studied adhesion protein, that given its strong evolutionary conservation is likely to have some implications for NCAM function, too. I support publication as is.

**Response:**Thank you very much for the positive comments. As suggested, we have changed Fig 5A to show non-tilted eye discs. The upper panels now show a control CyO sibling without the brain (which partially overlapped with the eye disc in the previous version). We also changed the images in the panels, previously showing section overlays, to display single optical sections (while the signal appears weaker, this provides a much cleaner image.

**Reviewer #2:** In the paper “Fasciclin 2 engages EGFR in an auto-stimulatory loop to promote imaginal disc cell proliferation in Drosophila” by Velasquez et al., the authors demonstrate a role for Fasciclin 2 in the regulation of cell growth and competition by positive regulation of EGFR signaling in imaginal discs, which is contrary to its previously demonstrated role in retinal differentiation. This paper provides an interesting and novel contribution to the field, with genetic and molecular biology evidence of the interaction between Fas2 and Egfr. This manuscript would be a good fit to PLOS Genetics after a more thorough exploration of pathways that are involved downstream of cell autonomous and cell non-autonomous effects. In addition, although the authors did a remarkable job writing in their non-native language, a few revisions to the text are highly encouraged to improve readability – at times, thanks to the evaluation of so many different pathways, the reader gets slightly confused (for example, see “minor suggestion 2”).

Major suggestions:

1. On Fig 2E, slowing growth via the minute technique appears to partially rescue clone size, but not completely back to wildtype. Are there other effects that are not cell competition that could account for the incomplete rescue?

**Response:** We do not think there is a need to invoke an additional effect to explain the phenotype of the fas2 null clones. Cell competition outcome and clone size would strictly depend on the relative growth rates between Fas2-deficient Minute^+^ cells and the Minute heterozygous ones in the background. We have found that Fas2-deficient cells have a mitotic rate much slower than WT, of course this does not have to match that of the Minute heterozygous background cells (in fact it seems to be slower). On the other hand, it has been recently shown that a Fas2 deficiency in the Follicular epithelium of the ovary can cause the misplacement of one in every 150 cells (Cammarota et al., 2020 Curr Biol 30, 5049), such a rare occurrence may be compatible with our results (although we have not detected it in imaginal discs).

2. For the apoptosis analysis, the authors should take into consideration the growth size of WT clones made into a “DfH99/+” or “diap1(OE)” background. Since the “rescue” of clone sizes is so minute (and UAS-P35 doesn’t even rescue it), genetic background may be an issue. Also, if the authors truly believe that apoptosis is “a consequence and integral part in the process of cell competition” (page 7), then the conclusion that apoptosis inhibition barely affects clonal size would contradict the authors findings. The authors should address the discrepancy (perhaps by finding evidence of/citing cases of cell competition without any major cell death? Or removing the cell death data?).

**Response:** Cell death occurs in some cells in the clones and it can be detected with anti-cleaved Caspase 3. We have now included a new picture showing apoptosis in some cells in a fas2^–^ null cell clone (Fig. S1I). Moreover, as explained in the Results, many fas2^+^ twin clones lack the corresponding fas2^–^ null sibling clone. It is clear that apoptosis occurs in the fas2^–^ clones, but it is also clear that it is not the principal cause of the small size of the clones (which is the reduced proliferation). The issue of why many fas2^–^ clone cells can evade apoptosis is critical to understand the reason for analyzing JNK function in fas2^–^ clones. Indeed, our work suggests that it is the increased JNK activity in fas2^–^ clones, causing increased Yki expression, what allows fas2^–^ clones to survive. Note that our experiments also show that not any increased activity of JNK allows for survival of fas2^–^ cells, since a large increase of JNK activity (caused by the loss of 50% in the JNK repressor Puc strongly enhances apoptosis). On the other hand, we have deleted the word “integral” which seems to cause some semantic misunderstanding in the purpose of the sentence. The text now reads: “Apoptosis of slow proliferating cells is a consequence of cell competition”. Note that the sentence does not necessarily imply that there should be apoptosis of all fas2^–^ null cells. In addition, we have now modified Fig. S1J (Fig. S1I in the previous manuscript) to include control clones for UAS-diap1 and UAS-P35 expression in a fas2^+^ background. The data on fas2^–^ ; DfH99/+ clones growing in a heterozygous DfH99/+ background does not seem to require the control of DfH99/+ clones growing in the DfH99/+ background, since the genotype for DfH99 is not changed in the clones (DfH99/+ individuals are phenotypically WT and completely viable and fertile).

3. The JNK analysis is well made and very interesting, but the authors did not quantify the results obtained in Fig 4, which is needed in order to appreciate the strength of the rescue (particularly in Fig 4D). It would also be nice to quantify how often JNK activation is observed under the different reporters and conditions.

**Response:** We have now included the quantification of TRE-DsRed and puc-LacZ in the different fas2^–^ studied conditions: en-GAL4 imaginal discs, MARCM clones and M^+^ clones (see the new FigS2).

4. Do slow-growing clones adjacent to Fas2 deficient cells also have reduced EGFR/Erk signaling, or is the cell-non-autonomous growth deficit mediated by another pathway? When you restore the EGFR signaling or downstream effectors in Fas2- cells, what happens to the neighbor cells?

**Response:** We thank the reviewer for this question, as it has helped us add an important new result. As stated in the manuscript, the fas2^+^ twin control clones in the fas2^–^ null coupled-MARCM analysis grow normal and have a normal mitotic index (Fig 2E and G). It is only the rim of normal cells contacting the Fas2-deficient clones that show a reduced mitotic index (Fig 2 G). The rest of the fas2^+^ control clone has a normal mitotic index (Fig 2G). In other words, it is only the normal Fas2-expressing cells in close proximity to the fas2^–^ null clone which show a reduced mitotic index. As explained in the manuscript, this fits well with the Fas2 homophilic adhesion between cells being the responsible for the growth function of Fas2. Moreover, we have now included the quantification of the mitotic index in both the fas2^–^ null clones rescued by activated-Egfr and the rim of normal cells contacting the rescued clones. The results are very clear: there is a very strong and cell autonomous increase of the mitotic index in the fas2^–^ activated-EGFR clone, while the rim of normal cells around the rescued clone show the same reduced mitotic index than the cells surrounding fas2^–^ clones in the coupled-MARCM analysis (see new Fig. 5G and compare with Fig. 2G). This result neatly demonstrates the cell autonomy of the activated-EGFR rescue and, at the same time, strongly reinforces the idea that Fas2 homophilic binding is required for EGFR activation.

4. Do Fas2- M+ clones differentiate normally, or can the switch in Fas2 to EGFR- suppression be demonstrated in this tissue?

**Response:** As shown in Fig S1 G, fas2 M^+^ clones differentiate normal epidermis (albeit with a somewhat lower density of bristles typical of the fas2 null condition, see Garcia-Alonso et al., 1995; also compare with the male fas2^–^ side in the gynandromorph in Fig S1A). This LOF alteration is strongly consistent with a lowered EGFR function, and reciprocal to the extra-bristle phenotype of fas2^–^ activated-EGFR (see Fig 5 D) which is typical of an EGFR gain-of-function.

5. Finally, though very interesting, the authors’ conclusion that Fas2 is also a direct, cell-autonomous target of Egfr could also have another explanation. Is Fas2 a target of Yki? Does JNK inactivation upon Egfr activation in clones rescue the increase in Fas2 seen in Figure 7? These experiments would help elucidate if Fas2 levels in Egfr or yki GOF clones are cell-autonomously promoted by Egfr, or is a result of the JNK/Yki non-autonomous activation.

**Response:** We thank the reviewer again for this question that helps further clarify the interactions between Fas2, Egfr, JNK and Yki. As shown in the Results, Fas2 is over-expressed by over-expression of either Egfr or Yki (Fig 7 A, B). Fas2 expression is controlled by Egfr activity (Mao and Freeman, 2009; Fig S4). We see that Fas2-deficient compartments or cells increase JNK expression, and JNK increased levels cause an increase in Yki expression (visualized as an increase in ex-LacZ expression). On the other hand, it is known that over-activation of EGFR in combination with JNK activity can lead to Yki increase and over-growth (Yamamoto et al., 2017). We have now performed the important experiment the reviewer suggests to see if the over-expression of Fas2 in activated-EGFR cell clones requires JNK activity. We have used a Fas2::GFP protein trap (see the new Fig. S6A) to monitor Fas2 expression in the activated-EGFR clones. The over-expression of Fas2::GFP in these clones is dramatic (so much that to avoid over-saturation in the image the normal Fas2 expression is barely detectable, see the new Fig. S6B). Importantly, when blocking JNK expression in these clones with bskRNAi there is no change in the dramatic increase of Fas2::GFP (see new Fig. S6C, D). The results are clear: activation of the EGFR pathway directly causes a cell autonomous increase of Fas2 expression.

Minor suggestions:

1. Some of the green/red labels inside panels are hard to read (for example, Fig S1 E- G). The authors could place a black rectangle behind the labels, or move them outside the images.

**Response:** We have now outlined in white all labels for easier reading.

2. As an example of text that would benefit from some changes in flow/rewriting for clarity, on page 8 the authors start by describing how they will evaluate JNK activity, by mentioning the TRE and puc reporters. Then the authors start describing the results of the TRE results (Fig 3E), go back to explain the puc reporter, then mention results from the previous evaluated theme of apoptosis (caspase 3 stains, Fig 3D), to then finally start describing the puc reporter analysis (Fig 4A). The manuscript would benefit from having this section re-written.

**Response:** We see the issue the reviewer raises. We have now transposed Figs. E, F and G, H to give a clearer flow in experiment descriptions. The section now first describes en-driven fas2RNAi conditions with TRE and Puc reporters, and then fas2^–^ null clones with TRE and Puc reporters.

3. The authors should use RNAi identifiers other than BL number in figure legends, as the Bloomington stock center reserves the rights to remove or change numbers.

**Response:** Here we do not agree with the reviewer. We think the numbers of Bloomington lines are the most objective identifier (if we put some other name, we still must refer to the Bloomington identifier). We think it is more appropriate to simply maintain the BL number in each case.

4. Missing reference to figure 1G

**Response:** The text now reads: “The hypomorphic mutant combination and the specific expression of two different fas2 RNAis in the wing imaginal disc caused a graded size reduction of the adult wing (Fig. 1C, quantified in G).”

5. It’s not clear whether their Ns are number of clones, number of discs, or number of animals. In cases where clones/discs are counted, number of animals should also be identified.

**Response:** We have now specified the nature of N in the figure legends, and specified in the Experimental Procedures the calibration of the heat-shock time for the frequency of clones in imaginal discs and adult nota.

6. Fig S1 lists N of 2 for “fas2- M+”. If that was indeed the case, the authors should increase significantly the number of clones analyzed.

**Response:** The number of 1^st^ instar larva (LI) M+ fas2– null adult clones has been increased to 15.

7. Title of figure 3 should be more descriptive. For example, “loss of Fas2 cell autonomously and non-autonomously activates JNK signaling”

**Response:** We think a title like “JNK is de-repressed in fas2 LOF conditions” would lack the necessary novelty. Rather, we think the current figure title better encompasses all the different aspects of our analysis.

8. The final model would be more useful if it included elements of the pathways tested (EGFR, JNK, etc).

**Response:** We agree. Fig. 7E now presents an additional schema showing the functional interactions between Fas2, EGFR and JNK revealed in our study. The figure legend now gives a summary explanation of the different interactions.

**Reviewer #3:** Previous studies have revealed interactions between the cell adhesion molecule Fas2 and the EGFR activity during morphogenesis. Thus, fas2 gain of function has been shown to promote EGFR activity during axon growth. In contrast, fas2 loss of function has been shown to cause de-repression of EGFR during retinal differentiation. In addition, experiments in the follicular epithelium of the Drosophila ovary has also revealed a role for Fas2 on regulating cell proliferation. These studies have shown that fas2- follicle cell clones displayed a 2-fold increase in BrdU incorporation and in cells in S-phase compared to controls. In addition, while wild type follicle cells stop dividing by stage 6, elimination of fas2 function resulted in the presence of PH3+ cells in later stages and in a 2-fold increase in the number of Cyclin E positive cells, showing that Fas2 represses follicle cell proliferation. All these results have led to suggest that the cell adhesion molecule Fas2 can regulate epithelial cell proliferation and dynamics acting not only as a localized scaffold but also by communicating signals to the nucleus. In this work, the authors analysed the role of Fas2 in another epithelial context, the Drosophila imaginal discs. In contrast to previous results mentioned above, here, the authors find that Fas2 loss leads to reduced proliferation. In addition, they find that fas2 mutant cells show decreased EGFR activity. In fact, based on genetic interactions and rescue analysis, they propose that Fas2 acts via the EGFR pathway to control cell proliferation. They further show that Fas2 and EGFR co-precipitate in cultured HEK293 cells. They also find that EGFR activity promotes the autonomous expression of Fas2, leading them to propose the existence of a feedback loop between Fas2 expression and EGFR activity in wing imaginal disc cells. Finally, they find that Fas2 loss induces increased JNK and Yki activity.

In general, I think there are some interesting results that challenge previous reported roles of Fas2 in cell proliferation and its interaction with other pathways, such as the EGFR, JNK or Hpo. However, I find the work still at a preliminary stage, there are serios problems with some of the results, as they are presented (see below, major concerns) and more work needs to be done to properly address the role of Fas2 in cell proliferation in the imaginal discs and to explain why Fas2 plays opposite roles in different cellular contexts. In summary, part of the analysis seems to me at a preliminary stage and several of the conclusions reached are not based upon a rigorous analysis. Therefore, I do not consider this work of enough scientific value as to merit publication in PLoS Genetics at its present stage.

Major concerns:

1. The authors use hypomorphic conditions and fas2 RNAis to knockdown fas2 expression in imaginal discs. However, it is not clear to me to what extent these two conditions reduce fas2 levels. Antibody staining with anti-Fas2 antibody would resolve this small issue.

**Response:** We are surprised by this statement. As stated several times in the main text and in the Figure legends, all coupled-MARCM and MARCM analyses in imaginal disc clones and adult epidermal clones have been done with the null mutation fas2eB112.

2. To confirm that Fas2 is required for cell proliferation, the authors generate Fas2 deficient clones using the Minute technique. They claim fas2-Minute+ clones displayed normalized size in both the wing disc and the notum. However, while clone size is similar to controls in the notum, this is not the case in the wing disc, where the size of the fas2-Minute+ clones are slightly bigger than fas2- clones alone and much smaller than fas2+ clones. Thus, to me, in contrast to what authors propose, this data suggests that Fas2 might be regulating something else besides cell proliferation in the wing disc cells.

A comment to add here is that, while the data for fas2+ or fas2- clones in the notum is represented as a dot plot, this is not the case for fas2-Minute+ clones. This makes difficult to properly assess the rescue.

**Response:** It is important to remark that it is the difference between the proliferation rate between the Minute+ fas2^–^ null cells and the fas2^+^ Minute heterozygous cells what determines which ones would grow more. M^+^fas2^–^ clones do not necessarily have to match the growing rate of Minute heterozygous cells, and therefore they do not have to necessarily reach the size of WT clones when growing in a Minute heterozygous background (although as can be seen in the dot plot in Fig 2E, the largest sizes of M^+^ fas2^–^ null clones in the wing imaginal disc are well within the range of WT clones). Rather, on average fas2^–^ null cells seem to grow at a somewhat slower rate than the Minute heterozygous cells. The important point here is: Why fas2^–^ cells can evade apoptosis if they proliferate even slower than Minute heterozygous cells? This question is what drove the following analysis on the role of JNK in fas2^–^ cells. On the other hand, the dot plot of fas2^–^M^+^ clones induced in 1^st^ instar larva in Fig. S1J (Fig. S1I in the previous version) has been updated to include 15 clones. The results clearly indicate that fas2^–^ null clones grow much better in a Minute heterozygous background, confirming that fas2^–^ cells have a slow proliferation rate. Note that the slow proliferation rate of fas2^–^ null cells is independently confirmed by their reduced mitotic index when compared with their fas2^+^ sibling clones (Fig. 2G).

3. To study the contribution of apoptosis to the fas2 phenotype, the authors test if suppression of apoptosis rescues the fas2- clones. They found this was the case, although slightly, and in certain conditions. However, this is only tested in the notum and not in the wing disc. Thus, I think the authors should also test the effect of inhibiting apoptosis in the wing disc. In general, I think the author switch between the wing disc and the notum to test different aspects of Fas2 function and I believe they should test all parameters in both contexts, as it is not clear, at least to me, that fas2- clones behave the same in both cellular contexts, since i.e., as mentioned above, fas2- Minute+ clones do not behave the same in the notum, nearly complete size rescue, compare to the wing disc, slight size rescue.

**Response:** Apoptosis is normally caused by the activation of JNK (bsk). Fig. 3C clearly shows that bskRNAi expression does not suppress the phenotype of fas2^–^ null clones. Therefore, the small size of fas2^–^ clones is not primarily caused by apoptosis. Both, imaginal disc clones and adult notum clones, in DfH99/+ individuals or expressing UAS-diap1, show the same: that apoptosis, albeit present to some extent in the fas2^–^ null clones (see the loss of many fas2^–^ twins in Fig. 2E, and the new Fig. S1I), it is not the cause of their small size. The message is that many Fas2-deficient cells, although growing slower than their normal neighbors, can somehow evade apoptosis. This is an important point because it is the reason to analyze the involvement of the JNK pathway in their survival. In summary, fas2^–^ null clones behave similar in all imaginal disc contexts, wing, eye and leg discs showing loss of growth (see Fig. 2A). This is quite different in abdominal histoblasts (a feature diagnostic of classic cell competition, see Fig. S1C) and the brain (see Fig. 2C).

4. Next, the authors analysed the involvement of the JNK pathway in the fas2 loss of function phenotype, by inhibiting its activity in fas2 mutant cells. They found that expression of an RNAi against the JNK basket did not rescue the size of the fas2- clones, suggesting little if any involvement of the JNK pathway in fas2 loss of function.

**Response:** This statement is unclear to us. We may say “suggesting little if any involvement of the JNK pathway in promoting apoptosis in the fas2 loss of function”, but for sure we would not say that JNK does not have any function in the fas2 loss condition. The message is exactly the opposite: JNK has an important function in fas2^–^ cells, but it is not to promote apoptosis. The level of JNK expression in the fas2^–^ null clones is critical to tilt the balance towards survival or towards apoptosis, as the experiment with puc-LacZ shows (as explained in the Results: Puc is a feed-back repressor of JNK and puc-LacZ causes a null mutation in the puc gene). We would state that the activation of the JNK pathway is not the primary cause of the reduced size of fas2^–^ clones. On the contrary, as presented in the manuscript, the JNK pathway seems to be a compensatory attempt in fas2^–^ cells to overcome the EGFR activity deficit by increasing Yki expression.

5. To further test the implication of the JNK pathway, the authors tested the expression of two downstream targets of this pathway in fas2- clones. Surprisingly, there is a big difference in the induction of the 2 downstream targets of the JNK pathway tested, TRE-DsRed and puc-lacZ. Thus, while there is a high increase in the levels of puc-lacZ in fas2 RNAi conditions, TRE-DsRed seems to be only slightly increased. In addition, levels of the two reporters have not been properly quantified. Furthermore, I think authors should provide an explanation for the difference between the expression of the two targets. In addition, the authors claim that a reduction in the dose of puc, as it happens in a puc-lacZ background, leads to widespread apoptosis and strong alterations in the A/P boundary. However, this is only observed in panel B of Fig.4, while the discs shown in panels A and C of this figure, besides having the same genotype, do not show these strong defects. Thus, how often are the defects shown in Fig4B observed? How strong is this interaction? Finally, with respect to this section, I am a bit confused about the contribution of the JNK pathway in the fas2 phenotype, I am not sure whether the authors relate it to apoptosis, although there is no sign of apoptosis in fas2-deficient clones, or growth, which is not clear to me what they mean with growth as they have shown fas2- cells do not change their size. In addition, as bsk RNAi is unable to rescue the fas2 mutant clone size, what is the functional meaning of the elevated expression of the JNK targets in the absence of fas2?

**Response:** We have now included the quantification of TRE-DsRed and puc-LacZ signal in the different fas2^–^ LOF conditions (en-GAL4 wing discs and MARCM fas2^–­^ null clones, new Fig. S2). As the reviewer correctly points out, there is a big difference between the expressions of TRE-DsRed and puc-LacZ reporters. As explained in the text, the reason for this seems obvious. As indicated by the TRE-DsRed expression, Fas2-deficient cells have a derepression of JNK. On the other hand, the puc-LacZ insertion causes a null mutation in the puc gene. Since Puc is a feedback repressor of bsk, the loss of 50% of Puc in the fas2^–^ cells causes an additional derepression of the JNK pathway on top of the de-repression caused by the fas2^–^ condition. This is the reason for the strong increase in JNK expression, and the associated JNK mediated apoptosis in fas2^–^ compartments when harboring the puc-LacZ insertion. This effect can be clearly seen in the anterior/posterior compartment border in all panels Fig. 4 A, B and C (see the outline of the en-driven GFP expression in the three panels). The A/P compartment border is normally a continuous wavy line. In the three panels, it is clear that the A/P boundary is compromised with frequent indentations (note that we have included the A/P outline because the DAPI signal partially masks the GFP in the RGB picture). These results lead us to an important conclusion: The level of JNK activity determines the balance between compensatory growth/ apoptosis to tilt towards survival or towards apoptosis (with low/medium levels of JNK activity promoting survival and high levels promoting apoptosis).

6. Then they test the contribution of EGFR activity to the fas2 loss of function phenotype. In order to do this, they again use the eye disc and the wing disc to test different things. On one hand, they test EGFR activity in fas2- clones in the eye disc, while, on the other hand, they test the ability of activated EGFR pathway to rescue fas2 loss of function in the wing disc. As mentioned above, I think they should all be tested in the same cellular context. In addition, pictures showing EGFR activity are not of enough quality. Thus, even though the internal control for ppERK levels, the ring gland, show similar levels in both experimental and control conditions, which it is in any case difficult to appreciate due to oversaturation of the signal, the general levels of ppERK in the eye disc outside the clones seems much lowered in the experimental condition compared to the control. In addition, there are many GFP positive cells that do not show changes in anti-ppERK staining. Thus, I believe this result, as it is presented, is not conclusive.

**Response:** There seems to be several misunderstandings in this statement. The ey>FLP experiment causes *all* cells in the eye-antenna disc to express GFP and fas2RNAi (there are no clones here), while the CyO sibling controls do not express either. The eyeless promoter is expressed in the eye-antenna imaginal disc and the optic lobe, but not in the Ring Gland (this is the reason for showing the Ring Gland of the same individual as internal control). The imaginal disc proliferation requirement for Fas2 is similar in the eye disc and the wing disc (see Fig. 2 A, B and C). The reason of using the eye disc is that the MAPK (pp-ERK) staining is easier to see and compare with the neighbor Ring Gland (which also expresses high levels of pp-ERK) than using wing discs. We have now substituted the pictures in Fig. 5A (which were confocal overlays) by single optical sections to avoid the presence of noise and background cells in the images. We have also substituted the control disc to avoid any overlap between the disc and the brain.

7. In the next section, the authors analyse the relationship between Fas2 and the Hippo pathway. In the follicular epithelium it has been shown that Fas2 tumors are suppressed by Wts overexpression, indicating that Fas2 signals through Wts to repress proliferation, although in this case Ex seems to play a minor role. Similarly, here, the authors show an upregulation of Yki activation in fas2- clones. As this is reverted upon JNK repression, the authors propose that indirect JNK activation by loss of fas2 promotes Yki activation. However, they do not find any effect on fas2 loss of function phenotype when suppressing wts. This questions the functional meaning of the elevated expression of ex-lacZ in fas2 clones. I would also be interested to know whether overexpression of wts would rescue loss of fas2 in the wing disc, as it does in the follicular epithelium.

**Response:** The contradiction between the phenomenology in imaginal discs and the follicular epithelium in the ovary is only apparent. We use ex-LacZ here as a reporter of Yki activity, as it is standardly used. Its increase over the normal level indicates that Yki is somewhat over-activated, and, as the reviewer correctly says, dependent on JNK activity (which is increased by the fas2RNAi condition). Thus, it may initially seem contradictory with previous reports on the interaction between Fas2 and the Hippo pathway. However, this is not the case. In this manuscript we are analyzing the role of Fas2 at low and medium levels of expression (those occurring during imaginal disc growth) but Fas2 behaves as a repressor of EGFR activity at high expression level (the expression level in pre-differentiating and differentiated cells). It would be in the context of high Fas2 expression where the phenomenology described by the reviewer would fit better. We invite the reviewer to see our prepublication in bioRxiv, <http://dx.doi.org/10.1101/356196>, as well as Yamamoto et al. (Nature 542, 246; see Fig. 4R) for a plausible scenario where the role of Wts in EGFR de-repression conditions (like those caused by a Fas2 deficiency in scenarios that require high expression levels of Fas2 to repress EGFR activity) would best fit.

In addition, we have carried out the analysis suggested by the reviewer: over-expression of Wts on fas2RNAi FLPOUTs. We find that 1^st^ instar UAS-wts16B FLPOUT clones appear at a frequency of 17/82 in the wing imaginal disc, and have a size not much smaller than WT clones. Instead, UAS-fas2RNAi#34084 FLPOUT clones are infrequent (3/95) and are very small. We did not find any UAS-wts16B UAS-fas2RNAi#34084 FLPOUT clones in 53 wing imaginal discs. Therefore, as expected, the repression of growth caused by fas2RNAi plus wts over-expression are either additive or synergistic.

8. Finally, the authors show that Fas2 physically interacts with EGFR in HEK293 cells. This is a heterologous system and this interaction might not be maintained in Drosophila imaginal disc cells. Could the authors test this interaction in Drosophila S2 cells? In addition, if these two proteins interact physically they should co-localize. The authors could test this by immunostaining wing discs from available transgenic flies carrying a superfolder-GFP inserted into the EGFR locus with anti-GFP and anti-Fas2 antibodies. Here, it is also worth mentioning that when analysing the ability of suppressing JNK on the observed activation of the Hpo pathway upon depletion of fas2, the number of samples analysed is only five. I do not think this sample is big enough as to propose a statistically significant difference. This should be better analysed by increasing sample size.

**Response:** First, we do not agree with the assessment that Drosophila S2 cells are better suited for this analysis than HEK293 cells. The vertebrate cells seem to be a better system to avoid possible Fas2 interactions with modulators of the Fas2/EGFR interaction in Drosophila (like CG15630 and CG 33543). Since CG15630 and CG33543 may block or interfere in the interaction between Fas2 and EGFR (see our prepublication in bioRxiv, <http://dx.doi.org/10.1101/356196>), the vertebrate system seems a more sensible choice. Second, As suggested by the reviewer, we now show the colocalization of Fas2 and EGFR in imaginal disc cells (see new Fig S5A). We have now increased the number of imaginal discs in Fig. S4 (old Fig. S3).

Minor comments

1. The authors refer to Fig.2B, C when talking about the rescue of fas2- clones with either Fas2GPI or Fas2TRM, however these panels only show Fas2GPI.

**Response:** We have now included the quantification of the rescue of fas2^–^ null MARCM clones with both Fas2GPI and Fas2TRM in Fig. S1J (old Fig. S1I).

**LIST OF CHANGES**

**Manuscript**

Pag. 5, line 16: inserted “quantified in”

Pag. 6, line 12: removed “both at the same time”, inserted “or”, removed Fas2TM, inserted Fas2TRM; line 13: inserted “Fig. S1J”; line 22: removed I, inserted “H, J”

Pag. 7, line 3: removed “and integral part in the process”; line 5: inserted “only”; line 8: removed “I”, inserted “J”, inserted “Apoptosis was found in a fraction of cells in fas2– cell clones (Fig. S1I)”; line 11: inserted “induction of fas2– cell clones in a heterozygous”; line 12: removed “the presence of”, inserted “background”, removed “slight but statistically”, inserted “barely”; line 13: removed “but”, inserted “and”; line 14: removed “I”, inserted “J”; line 22: removed “J”, inserted “K”

Pag. 8, line 6: inserted “Fig. S2A left”; line 13: inserted “Fig. S2A right”; line 19: removed “Strong”, inserted “Over-“; removed “puc-LacZ” inserted “the TRE-DsRed reporter”; line 20: inserted “Fig. S2B left”, transposed “Left, quantification of TRE-DsRed signal in *en-GAL4 UAS-GFP/TRE-DsRed (fas2^+^*) and *en-GAL4 UAS-GFP/TRE-DsRed; UAS-fas2^RNAi#34084^/+* (*fas2^–^­*) wing imaginal discs. The TRE-DsRed signal was amplified using an anti-RFP antibody, and the signal in the anterior compartment was subtracted to the signal in the Posterior compartment in each disc to normalize for differences in staining. Right, quantification of puc-LacZ signal in *en-GAL4 UAS-GFP/+; puc-LacZ/+* (*fas2^+^*) and *en-GAL4 UAS-GFP/+; UAS-fas2^RNAi#34084^*/*puc-LacZ* (*fas2^–^­*) wing imaginal discs. The signal in the anterior compartment was subtracted to the signal in the Posterior compartment in each disc to normalize for differences in staining. N is number of wing imaginal discs”; line 22: removed “puc-LacZ”, inserted TRE-DsRed”; line 24: removed “puc-LacZ”, inserted “a suppression in TRE-DsRed”, inserted “Fig. S2B left

Pag. 9, line 1: inserted “fas2– cell clones”; line 2 through 7: inserted “displayed a strong expression of puc-LacZ (Fig. 4G; Fig. S*2B* right). This *puc-LacZ* expression, apoptosis and clone growth was reverted to normal by the simultaneous expression of the Fas2^GPI^ isoform in the clones (Fig. 4D), showing that the extracellular part of Fas2 is sufficient to support the function of Fas2 on cell proliferation in imaginal discs and revert JNK activation. Expression of *puc-LacZ* was abolished when *fas2^–^* *M^+^* cell clones were induced in a *Minute* heterozygous background (Fig. 4H; Fig. S*2B* right), in addition”; line 11: removed “The over-activation of the JNK pathway in fas2– null cell clones and its corresponding reduction in fas2 M+ clones was confirmed using the TRE-DsRed reporter (Fig. 4G, H)”; line 17: removed “2”, inserted “3”; line 21: removed “2”, inserted “3”; line 24: removed “2”, inserted “3”

Pag. 10, line 8: removed”2”, inserted “3”; line 9: inserted “Expression of activated-EGFR in *fas2^–^* cell clones caused a significant increase in the mitotic index within the clone, while it remained low in the rim of Fas2-normal cells surrounding the clone (Fig. 5G, compare to Fig. 2G). This result is strongly consistent with the idea that Fas2 homophilic binding is required for promoting EGFR activity.”; line 14: removed “2”, inserted “3”; line 15: removed “2”, inserted “3”

Pag. 11, line 15: removed “3”, inserted “4”; line 21: removed “3” inserted “4”

Pag. 12, line 1: inserted “Fas2 and EGFR are co-expressed and colocalize in all cells of the growing imaginal discs (Fig. S*5*A).”; line 3: removed “4”, inserted “5B”; line 10: inserted “). It has been shown that EGFR over-activation and JNK activity can interact to promote Yki expression (see Yamamoto et al., 2017). To see if Fas2 over-expression in the EGFR over-activation condition requires the function of JNK, we generated *activated-EGFR bsk^RNAi^* cell clones in a Fas2::GFP protein trap background. *T*he suppression of JNK activity *in these clones* did not *reduce* the over-expression of Fas2 (Fig. S*6C, D*), demonstrating that expression of Fas2 is directly controlled by the EGFR signaling pathway.”

Pag. 13, line 20: removed “cell clones null for”, inserted “null cell clones”

Pag. 14, line 21: inserted “(see Fig. 7E for a summary of the functional interactions between Fas2, EGFR and JNK)”

Pag. 15, line 12: removed “i”, inserted “wa”

Pag. 16, line 11: removed “paracrine”; line 12: “this requirement”, removed “for”, removed “activity”, inserted “the enhanced expression of”; line 13: removed “depend on it”, inserted “be a compensatory response to the loss of EGFR activity (see Fig. 7E for a summary of interactions between Fas2, EGFR and JNK)”; line 14: removed “rather than on its autocrine function which promotes apoptosis”

Pag. 17, line 13: inserted “The Fas2::GFP line was a gift from Dr. Christian Klambt”; line 16: inserted “This yielded an average frequency of 1 clone *per* wing disc and 1 notum clone every 5 adult flies for *clones generated during* 1*^st^* instar larva. Clones were induced”; line 21: removed “*fas2TM*”, inserted “*fas2TRM*”; line 24: removed “Fas2

Pag. 19, line 15: inserted “or a Leica Thunder”; line 17: removed “and”, inserted “Images were”

Pag. 20, line 17: inserted “Christian Klambt for the fas2::GFP line”; line 23: removed “AEI/FEDER, UE;”, inserted “grant”, inserted “funded by MCIN/AEI/ 10.13039/501100011033 and by “ERDF *a* way of making Europe”;”

Pag. 29, line 5: inserted “Yamamoto M., Ohsawa S., Kunimasa K. and Igaki T. (2017). The ligand Sas and its receptor PTP10D drive tumor-suppressive cell competition. Nature 542, 246-250.”

Pag. 31, line 11: inserted “N is number of individuals”

Pag. 32, line12: inserted “N is number of individuals”; line 15: inserted “inserted “N is number of wing imaginal discs”; line 21: inserted “N is number of clones”

Pag. 34, from line 10 to page 35: transposition and insertion of panels descriptions “MARCM fas2^eB112^; TRE-DsRed/+ cell clones display expression of the reporter (arrow) as well as a weaker non-cell autonomous expression (arrowhead), indicating derepression of the JNK signaling pathway. Clone induced in 2^nd^ instar larva.

Slowing the proliferation rate of the cellular background (labeled with Ubi-GFP, using the Minute technique) reduces JNK derepression in the fas2^eB112^ M^+^ cell clones, as indicated by the expression of the reporter TRE-DsRed in only a few cells of the clones (arrow). Arrowheads point to non-cell autonomous expression of TRE-DsRed in the Minute/+ heterozygous background. Clone induced in 2^nd^ instar larva.

Compared with the fas2^eB112^ rescued clones (in D), fas2^eB112^; puc-LacZ/+ MARCM cell clones are extremely small and display a very strong expression of the JNK pathway reporter. Clone induced in 1^st^ instar larva.

Reducing the proliferation rate in the cellular background (labeled with Ubi-GFP, using the Minute technique) abolishes reporter expression in the fas2^eB112^M^+^; puc-LacZ/+ cell clones (devoid of GFP expression), indicating a strong reduction in the activity of the JNK pathway. Note that the simultaneous deficit of Fas2 and half of the normal dosage of puc prevents these cell clones to grow as large as fas2^eB112^ M^+^ clones with a normal dosage of puc. Clone induced in 1^st^ instar larva.”

Pag. 35, line 12: inserted “RG”; line 20: inserted “N is number of individuals”

Pag. 36, line 7: inserted “N is number of individuals”; line 23: inserted “N is number of clones”; line 24: inserted “The mitotic index (relative to WT, as in Fig. 2G) in fas2^–^ activated-EGFR clones is highly increased, while the 2-3 cell rim of fas2^+^ cells around the clone shows the same value than in the rim of fas2^+^ cells around fas2^–^ clones (compare with Fig. 2G). N is number of clones.”

Pag. 37, line 24: inserted “cell autonomous”

Pag. 38, line 1: inserted “Note that Fas2 over-expression exactly matches the contour of the clones”; line 18: inserted “Summary of functional interactions between Fas2, EGFR and JNK during imaginal disc growth. Top,”; line 21: inserted “The activity in the JNK pathway remains minimal. Bottom, a cell deficient for Fas2 lacks the corresponding homophilic interactions with their normal neighbors. In such an scenario, EGFR activity is low and also somewhat reduced in the Fas2-normal contacting cells (note that these still keep their normal Fas2 homophilic binding with other Fas2-normal cells). In the absence of Fas2 homophilic binding, cell to cell interactions promote the activity of JNK producing an increase in Yki expression. The negative feed-back on JNK provided by Puc is critical to prevent shifting the balance in JNK expression towards apoptosis.”

**Figures**

Fig. 4, swapping of panels G, H to E, F

Fig. 5, change of images in panel A by single optical sections; change of picture in top image to avoid overlapping with the Optic Lobe and correct position; insertion of new panel G (mitotic index in fas2– activated EGFR FLPOUT clones and the rim of normal neighbor cells)

Fig. 7, insertion of new scheme in panel E summarizing the functional interactions between Fas2, EGFR and JNK

**Suplementary Figures**

Fig. S1, new panel I to show apoptosis in some fas2– clone cells; insertion in panel J of quantitative data on fas2– null MARCM clones in adults and their rescue by the expression of the Fas2 GPI and TRM isoforms

Fig. S2, this is a new figure to show quantitative data of TRE-DsRed and puc-LacZ expression in en-GAL4 fas2RNAi wing imaginal discs, as well as in fas2_ null MARCM clones and the corresponding rescue using The Minute technique

Fig. S3, is the old Fig. 2

Fig. S4, the number of samples for controls and for fas2RNAi bskRNAi has been substantially increased, the level of significance of the rescue by bskRNAi on the expression of ex-LacZ in fas2RNAi has increased correspondingly

Fig. S5, this Figure now includes the colocalization of Fas2 and EGFR in wing imaginal discs in panels A

Fig. S6, this is a new Figure to show that Fas2 over-expression caused by the overactivation of EGFR is independent of JNK

**Supplementary Figure legends**

Pag. 2, line 11: inserted “Bar is 50 μm”; line 12: (I) Expression of cleaved Caspase 3 in *fas2^–^* null clones is limited to a fraction of cells (arrow). Bar is 50 μm.”; line 14: inserted “, and rescue of *fas2^–^* MARCM clones by expression of *UAS-fas2^GPI^* and *UAS-fas2^TRM^”*; line 16, inserted “grey bars”, removed “and 2^nd^ (LII) instar larva”; line 18, removed “grey bars”; line 21: removed “ also produced a slight but significant suppression in the phenotype”, inserted “little significant (depending on the statistical test used, t or Mann-Whitney)”; line 23, inserted “had a similar effect, while expression of *UAS-P35* had no effect.”, line 24: inserted N is number of clones.”

Pag. 3, line 3: inserted “N is number of individuals”; line 5: inserted new description of new Figure “**Fig. S2. Quantification of the expression of JNK activity reporters**

(A) Left, quantification of TRE-DsRed signal in *en-GAL4 UAS-GFP/TRE-DsRed (fas2^+^*) and *en-GAL4 UAS-GFP/TRE-DsRed; UAS-fas2^RNAi#34084^/+* (*fas2^–^­*) wing imaginal discs. The TRE-DsRed signal was amplified using an anti-RFP antibody, and the signal in the anterior compartment was subtracted to the signal in the Posterior compartment in each disc to normalize for differences in staining. Right, quantification of puc-LacZ signal in *en-GAL4 UAS-GFP/+; puc-LacZ/+* (*fas2^+^*) and *en-GAL4 UAS-GFP/+; UAS-fas2^RNAi#34084^*/*puc-LacZ* (*fas2^–^­*) wing imaginal discs. The signal in the anterior compartment was subtracted to the signal in the Posterior compartment in each disc to normalize for differences in staining. N is number of wing imaginal discs.
(B) Left, quantification of TRE-DsRed signal in *fas2^eB112^FRT19A; TRE-DsRed/; Tub-GAL4 UAS-GFP/+* MARCM null cell clones and *fas2^eB112^ FRT18A; TRE-DsRed/+* *Minute^+^* null cell clones induced in *fas2^eB112^ FRT18A/Ubi-GFP M(1)O^sp^ FRT18A; TRE-DsRed/+* wing imaginal discs. The signal in the background was subtracted to the signal in the clone in each disc. Right, quantification of puc-LacZ signal in *fas2^eB112^FRT19A; Tub-GAL4 UAS-GFP/puc-LacZ* MARCM null cell clones and *fas2^eB112^ FRT18A; puc-LacZ/hs-FLP* *Minute^+^* null cell clones induced in *fas2^eB112^ FRT18A/Ubi-GFP M(1)O^sp^ FRT18A; puc-LacZ/hs-FLP* wing imaginal discs. The signal in the background was subtracted to the signal in the clone in each disc. N is number of clones.

Pag. 4, line 1, removed “2”, inserted “3”; line 7: inserted “N is number of eye imaginal discs”; line 19: inserted “N is number of clones”; line 22: removed “3”, inserted “4”

Pag. 5, line 8, removed “does not modify the normal”, inserted “slightly increases”; line 9: removed “but”, inserted “it strongly”; line 10: inserted “N is number of wing imaginal discs”; line 12: removed “4”, inserted “5”; line 13: inserted “(A) Fas2 and EGFR are expressed by all cells in imaginal discs. A Fas2::GFP protein trap (Neuert et al., 2019) shows colocalization with EGFR (ImageJ Colocalization plug-in, Pearson´s correlation: 0,3146).”; line 20: inserted new description of new Figure “**Fig. S6. Fas2 expression is directly regulated by EGFR**

(A) A Fas2::GFP protein trap line shows expression of Fas2 in all cells of imaginal discs, with a maximum in differentiating retinal cells.
(B) FLPOUT *UAS-LacZ* clones (red signal) expressing activated-EGFR display a dramatic increase in Fas2::GFP expression. Note that the saturation of the Fas2::GFP expression prevents the visualization of the normal Fas2::GFP expression in the other cells of the wing disc (compare to Fig. 7A which is stained with the 1D4 antibody that only recognizes the TRM isoforms of Fas2).
(C) Wing imaginal disc FLPOUT *UAS-LacZ* clones (red signal) expressing activated-EGFR plus *bsk^RNAi314767^* display a Fas2::GFP signal similar to activated-EGFR clones.
(D) Eye imaginal disc FLPOUT *UAS-LacZ* clones (red signal) expressing activated-EGFR plus *bsk^RNAi#31476^* display a similar strong Fas2::GFP signal. Note that the saturation of the Fas2::GFP signal only permits a very faint visualization of the normal Fas2 peak of expression in the differentiating retina.

**Supplementary Experimental Procedures**

Pag. 1, line 22: inserted “*y w hsp70-flp; Act5C-FRTy^+^FRT-GAL4 UAS-LacZ* “line 23, inserted “*y w hsp70-flp; Act5C-FRTy^+^FRT-GAL4 UAS-LacZ*/SM6a-TM6B/*UAS-bsk^RNAi31476^”*; line 24: inserted “*y w fas2::GFP; UAS-λEGFR”*
